# Syncopal Episodes in a Patient With a Large Hiatal Hernia: Exploring a Rare Cause-and-Effect Relationship Between Two Common Pathologies

**DOI:** 10.7759/cureus.77771

**Published:** 2025-01-21

**Authors:** Husnain R Ali, Angelo Materia, Irteza Afzal, Zunair I Afzal, Francis J O'Neill

**Affiliations:** 1 Medicine, American University of the Caribbean School of Medicine, East Meadow, USA; 2 Internal Medicine, Nassau University Medical Center, East Meadow, USA; 3 Cardiology, Nassau University Medical Center, East Meadow, USA

**Keywords:** guillain-barré syndrome, left atrium compression, mechanical compression, postprandial syncope, s:hiatal hernia, unusual cause of syncope, vasovagal syncope (vvs)

## Abstract

This report presents a case of an 80-year-old Caucasian female with a history of hypertension, deep vein thrombosis (managed with warfarin), and Guillain-Barré syndrome, who experienced a syncopal episode. The diagnostic workup included continuous cardiac monitoring, a transthoracic echocardiogram, and orthostatic vital measurements to identify potential causes of syncope. After ruling out more common etiologies, the syncopal episode was suspected to result from mechanical compression of the left atrium by an untreated hiatal hernia. While surgical correction was considered, the patient declined due to her age and the associated postoperative risks. Consequently, supportive management became the focus of care. This case highlights the importance of comprehensive evaluations for patients with recurrent syncope and the need to provide appropriate lifestyle recommendations when surgical intervention is not pursued.

## Introduction

Syncope, defined as a temporary loss of consciousness (LOC) caused by inadequate blood flow to the brain, accounts for approximately 1.0-1.5% of all ED visits [1]. It is traditionally classified by the underlying mechanism responsible for the patient’s symptoms. The primary etiologies of syncope include neurological, cardiac, orthostatic, and vasovagal causes [2].

Neurally mediated syncope, also referred to as vasovagal or situational syncope, occurs in the absence of heart disease and is triggered by factors such as prolonged standing, eating, vomiting, and the presence of prodromal symptoms like diaphoresis, dizziness, and nausea. Orthostatic hypotension involves volume depletion, primary autonomic failure, or the effects of polypharmacy, which require careful medication reconciliation. Cardiac syncope is often associated with symptoms such as palpitations, arrhythmias, diaphoresis, chest pain, or underlying infiltrative heart disease [1].

In this case report, the patient presents with a hiatal hernia, a condition characterized by the protrusion of the upper portion of the stomach and/or other abdominal organs through an anatomical defect in the diaphragm. Hiatal hernias are classified into four major types based on the extent of gastroesophageal displacement through the diaphragm. The leading theory of causation involves muscle weakness combined with a loss of flexibility and elasticity, resulting in hernia formation. Common symptoms include heartburn and regurgitation. Hiatal hernias are typically diagnosed through endoscopy, particularly when classic symptoms of reflux or dysphagia are prominent, and esophagography, which provides detailed imaging of the hernia and its thoracic location [3].

## Case presentation

**Figure 1:**
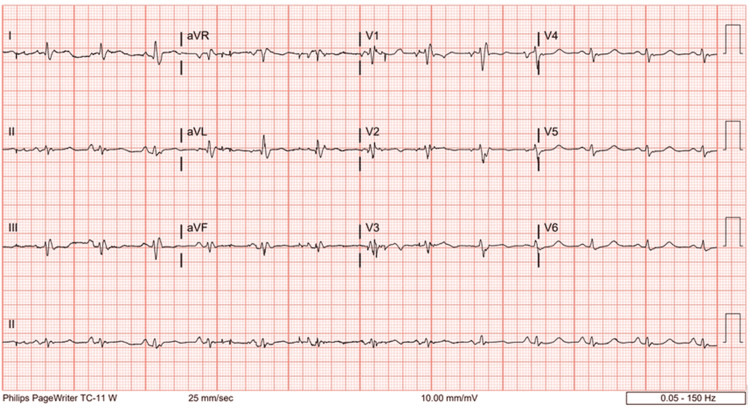
EKG demonstrating NSR with a nonspecific intraventricular conduction delay The PR interval is 161 ms, HR is 78 beats per minute, and QTc is 457 ms. NSR, normal sinus rhythm; QTc, corrected QT interval

The patient was an 80-year-old Caucasian female with a past medical history of hypertension (on doxazosin) and deep vein thrombosis, status post inferior vena cava filter and on warfarin. She ambulated with a walker at baseline due to her history of Guillain-Barré syndrome (GBS). She was brought to the hospital via ambulance for a sudden LOC after dining at a restaurant, with no traumatic head injury. After about one to two minutes of LOC, she regained consciousness and vomited. The patient adamantly denied tongue-biting or tonic-clonic movements. Further history revealed a similar LOC episode associated with vomiting occurring in 2022, which prompted a visit to the hospital. She was diagnosed with a hiatal hernia but declined surgical interventions at that time, citing her age and concerns about potential post-surgical complications. Family history was noncontributory, and her social history was negative for tobacco, alcohol, or drug use (Figure 1).

The patient’s body proportions indicated she was overweight, with a weight of 170 lbs, a height of 170 cm, and a BMI of 26.7 kg/m². The remainder of the physical exam was unremarkable, except for a facial droop upon ED evaluation, which led to a stroke code being called. The National Institutes of Health Stroke Scale score was 0, and the Glasgow Coma Scale was 15 throughout the hospital course. The patient was hemodynamically stable, with blood pressures ranging from the 120s to 130s/70s to 80s mmHg and heart rates in the 70s to 80s bpm. Neurology was consulted due to the patient’s history of GBS. During the assessment, the patient denied symptoms of weakness or vomiting, suggesting that GBS was not acutely exacerbating her current condition. The remainder of the vitals and oxygen saturation were within normal limits, as outlined in Table 1.

**Table 1:** Lab values during hospital admission ALP, alkaline phosphatase; ALT, alanine transaminase; AST, aspartate transaminase; BNP, B-type natriuretic peptide; eGFR, estimated glomerular filtration rate; MCHC, mean corpuscular hemoglobin concentration; MCV, mean corpuscular volume

Lab test	Value	Normal range
Troponin I – high sensitivity	6.48 ng/L	0-38.67 ng/L
Creatinine kinase	87 U/L	34-145 U/L
Lactate level	0.8 mmol/L	0.5-1.6 mmol/L
ProBNP	47 pg/mL	0.0-99.0 pg/mL
Comprehensive metabolic panel		
Sodium	140 mmol/L	136-145 mmol/L
Potassium	4.9 mmol/L	3.5-5.1 mmol/L
Chloride	102 mmol/L	98-107 mmol/L
Bicarbonate	34 mmol/L	20-31 mmol/L
Anion gap	4	5-15
Glucose, random	129 mg/dL	136-145 mg/dL
Urea nitrogen	15 mg/dL	9-23 mg/dL
Creatine	0.7 mg/dL	0.6-1.0
eGFR	>60 ml/min/1.73²	
Calcium	8.9 mg/dL	8.3-10.6 mg/dL
ALT	14 U/L	7-40 U/L
AST	15 U/L	13-40 U/L
ALP	73 U/L	43-86 U/L
Total bilirubin	0.4 mg/dL	0.2-1.1
Total protein	5.9 g/dL	5.7-8.2 g/dL
Albumin	3.2 g/dL	3.4-5.0 g/dL
A:G ratio	1.19 mmol/L	0.60-1.50
Magnesium	2.0 mg/dL	1.6-2.6 mg/dL
Phosphorus	4.0 mg/dL	2.4-5.1 mg/dL
White blood cell	7.73 K/mm³	4.50-11.00 K/mm³
Red blood cell	3.73 K/mm³	4.20-5.40 K/mm³
Hemoglobin	9.3 g/dL	12.0-16.0 g/dL
Hematocrit	31.30%	38.0-47.0%
MCV	83.9 fL	80.0-96.0 fL
MCHC	29.7 g/dL	32.0-36.0 g/dL
Red distribution width	15.30%	11.0-15.8%
Platelets	46.8 K/mm³	150-450 K/mm³

Lab work on admission was largely benign, with the CBC revealing mild anemia (hemoglobin 10.1 g/dL, hematocrit 34.2%). The comprehensive metabolic panel, magnesium levels, and lipid panels were within normal limits. The initial INR was 1.8, with a prothrombin time value of 20.3 seconds, indicating the need to adjust warfarin to therapeutic levels (INR: 2-3). Cardiac markers, including high-sensitivity troponin and creatine kinase, were negative. The patient initially had an elevated lactate level, which eventually normalized. The laboratory data did not provide significant contributions to clinical management, with the focus shifting to optimizing anticoagulation therapy while addressing other aspects of the patient's care. The remainder of the patient’s labs are described in Table 1.

**Figure 2:**
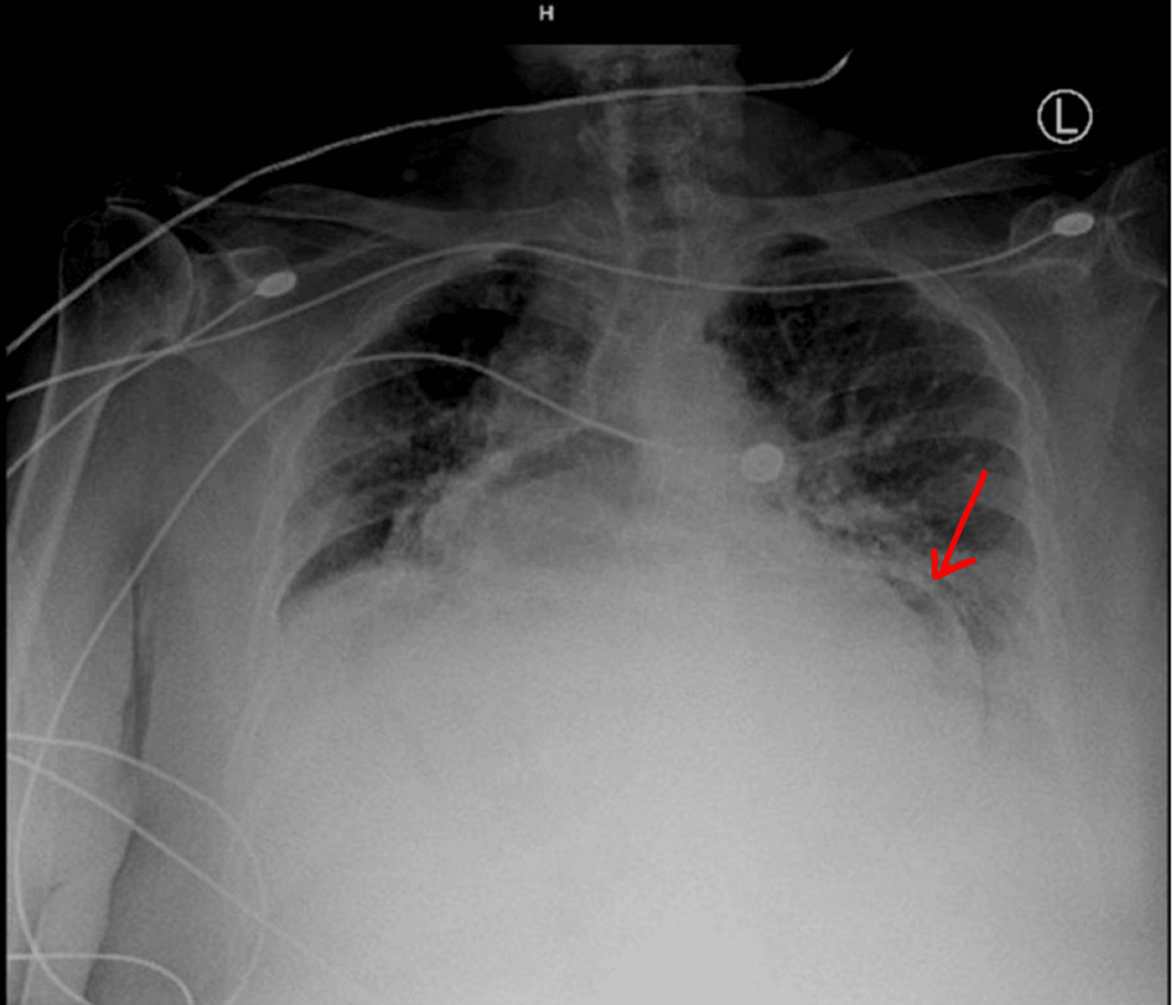
Chest X-ray revealing pulmonary vascular congestion and bibasilar effusions The presence of air on the left side of the cardiac silhouette is highlighted by a red arrow, denoting the stomach. The positioning of the stomach within the thoracic cavity is indicative of a hiatal hernia.

**Figure 3:**
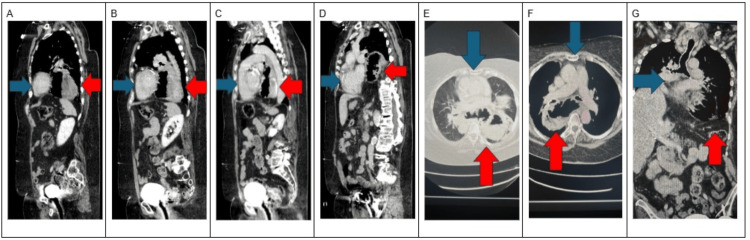
Imaging cuts illustrating a large hiatal hernia with compression of the left atrium: (A-D) Sagittal views highlighting the hernia. (E, F) Transverse views further demonstrating the anatomical relationship. (G) Coronal view showing the extent of the hernia. The red arrow indicates the stomach, while the blue arrow indicates the heart.

Imaging revealed a large hiatal hernia on abdominal X-ray and CT scans, with the entire stomach herniating into the lower thorax. Additionally, findings suggestive of compressive atelectasis were noted (Figure 2, Figure 3). An echocardiogram showed left ventricular wall thickening, with an estimated ejection fraction of 55-60% and grade I diastolic dysfunction. The cavity size and thickness of the right ventricle were within normal limits, while mild dilation of the left atrium was also observed. The mitral valve showed mild calcification of the annulus, moderate calcification of the leaflets, and trace regurgitation. These results indicated a degree of mild cardiac dysfunction.

The etiology of the syncopal episode was hypothesized to be primarily mechanical due to the proximity of the heart directly in front of the stomach. The patient was followed up by the surgical team, which discussed the possibility of surgical intervention to correct the defect. However, after extensive discussion, the patient declined surgery, citing her advanced age and the associated risks of such a procedure. Supportive care was recommended for the hiatal hernia, including a bowel regimen, a mechanical soft diet, and lifestyle modifications, such as consuming smaller, more frequent meals.

The remainder of the hospital course was uneventful, with the primary team focusing on assessing the patient for potential further syncopal episodes through a meticulous workup to exclude more common causes of syncope. This workup included continuous cardiac monitoring, which revealed no arrhythmia; measurement of orthostatic vitals, which showed no orthostatic hypotension; and imaging of the head, which did not reveal any intracranial causes for the syncope. The patient was sent home with a Holter monitor; however, she was lost to follow-up.

**Figure 4:**
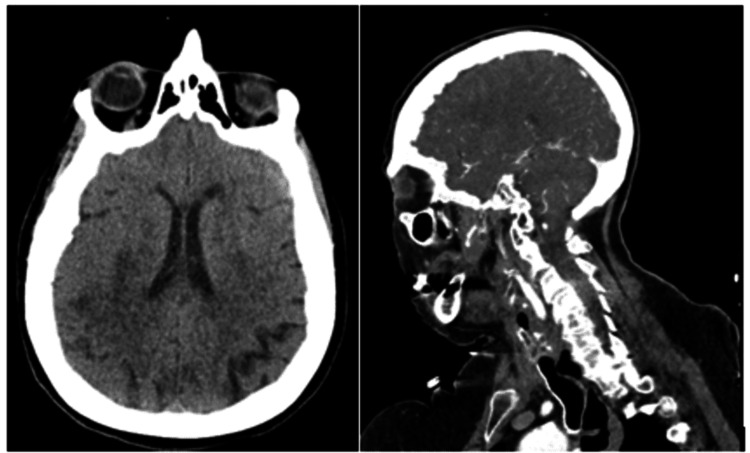
CT angiography of the head and neck The left panel shows the head, while the right panel displays the neck.

Further workup included a CT angiography of the head with contrast, which revealed no evidence of aneurysm, cranial stenosis, or vascular malformations (Figure 4). Neurology was consulted and agreed with the primary team’s assessment that there were no intracranial causes to explain the syncope.

## Discussion

This patient presented to the ED with a syncopal episode after consuming a large meal, which was not associated with seizure-like symptoms such as tongue biting or a post-ictal phase. Furthermore, lab results did not indicate epileptic activity, as lactate and creatine kinase were within normal limits, ruling out seizures as a cause of the syncopal episode. The patient’s disclosure of a hiatal hernia prompted in-house imaging, which revealed a severe hiatal hernia, with a large portion of the stomach located retrosternal to the heart. Therefore, the presence of a hiatal hernia in the context of a large meal may suggest that the effect of stomach expansion due to the bolus of food resulted in mechanical compression of the heart, causing left atrial outflow/inflow tract obstruction and thereby decreasing cardiac output, which resulted in the syncopal episode [4].

Symptomatic left atrial compression secondary to a hiatal hernia is an extremely rare pathology. A search on PubMed using the term “left atrial compression” yielded a total of 271 results. Among these, only 17 cases were identified where left atrial compression resulted from structures within the gastrointestinal tract. Of these 17 cases, 11 were specifically associated with hiatal hernias [5]. This suggests that this specific cause of syncope may be underrepresented, considering around one-third of the general population experiences syncope and 55-60% of patients over the age of 50 will have a hiatal hernia [2,6].

The context of eating a large meal may represent a less common etiology of vasovagal syncope, which could have potentially contributed to the overall LOC. The absence of “aura,” lateral-tongue biting, confusion, and palpitations suggests that seizure-induced syncope is less likely. During the patient’s hospital stay, she had continuous cardiac monitoring, which did not show any episodes of either bradycardia or tachycardia, thus making arrhythmogenic causes less likely. Furthermore, the presence of prodromal symptoms suggested a vasovagal etiology. Vasovagal syncope results in a profound decrease in cardiac output, primarily through increased vagal activity leading to decreased systemic arterial pressure in a phenomenon known as “vaso-depression.” This results in bradycardia, thus decreasing cerebral perfusion [7]. The patient’s advanced age may also have influenced the presentation, as studies using a tilt-table test showed higher rates of vaso-depression in older individuals. This is likely due to age-related neurological degeneration that causes autonomic dysfunction [8].

A brief literature review also suggests that a past medical history of hiatal hernia can be a precipitating factor for syncope. One patient is believed to have experienced syncope secondary to physical compression of the heart via a hiatal hernia, with symptoms occurring after swallowing. The esophageal hernia in this patient was thought to have contributed to the syncopal symptoms due to extrinsic compression of the heart. Surgical correction was ultimately performed, relieving this extrinsic compression and alleviating the patient’s symptoms [9]. Another case involved a 48-year-old man with a known history of a hiatal hernia, who experienced transient syncopal episodes during meals, believed to be caused by deglutition syncope. The main trigger in this case was swallowing, which resulted in increased intrathoracic pressure, thus compressing the vagus nerve [10]. An 82-year-old woman presented with repeated postprandial syncope, which had increased in incidence in recent years. After excluding cardiovascular, cranial nerve, and endocrine causes, it was found that a large hiatal hernia resulted in a protrusion of the transverse colon and part of the pancreas into the mediastinum, creating a mass effect that subsequently compressed the left atrium [11]. Another case involved a Type III hiatal hernia with posterior compression of the left atrium in a 92-year-old man who had experienced recurrent episodes of syncope for the past four weeks, consistently triggered by large meals. This patient’s presentation most closely matched the patient in our case report. Posterior heart compression of the left atrium, causing mechanical outflow obstruction; simultaneous activation of the Bezold-Jarisch reflex in response to increased cardiac pressures; and vagal stimulation during swallowing were all contributing factors to syncope in this patient [12].

**Figure 5:**
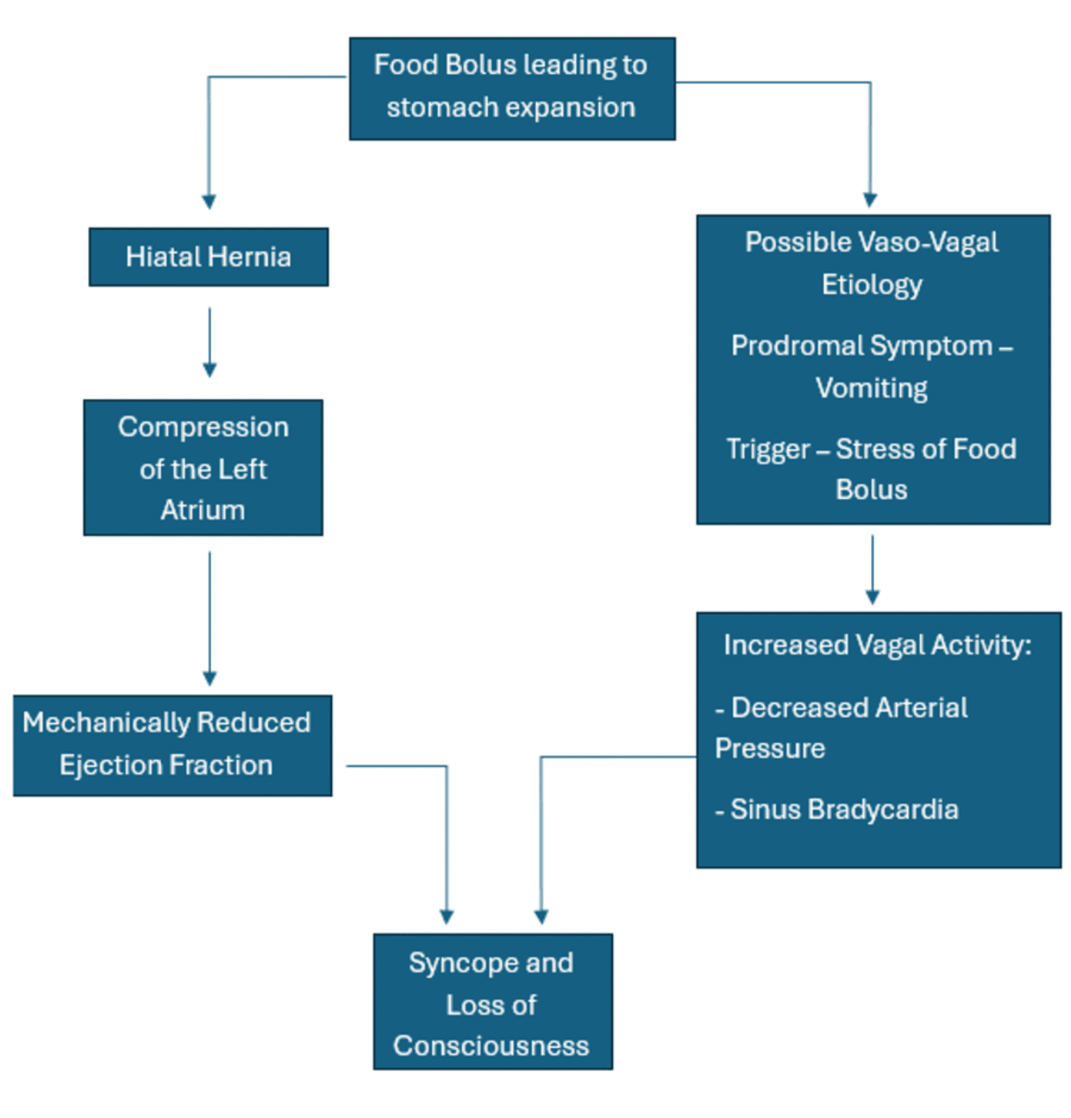
Pathophysiology elucidating the cause of syncope due to hiatal hernia

Based on these cases and on the presenting symptoms of our patient, we hypothesize that the underlying pathophysiology is likely due to three main factors: compression of the left atrium, thereby impeding blood flow to the rest of the body; increased vagal nerve stimulation leading to sinus node slowing; and abnormal reflex responses such as the Bezold-Jarisch reflex [13]. This reflex involves a complex interplay between cardiac mechanoreceptors and the autonomic nervous system. The mechanoreceptors in the inferolateral wall of the left ventricle, atria, and pulmonary artery are thought to be integral to the pathogenesis of this response, as they respond to stress and noxious stimuli, such as those caused by a hiatal hernia [14]. Contributing factors of syncope secondary to hiatal hernias are outlined in Figure 5.

Management of hiatal hernia-related syncope is primarily nonpharmacological. The preferred treatment is usually surgical correction of the defect. If the patient refuses surgery, the management approach should include the following:

First-line therapy involves patient education and lifestyle modifications, such as recognizing and avoiding triggers and situations that may provoke syncope. Active counter-pressure maneuvers, like handgrip and leg crossing, should be initiated. These maneuvers are believed to help inhibit vasovagal syncope by increasing venous return [15]. Additionally, the patient should be encouraged to reduce portion sizes and increase meal frequency throughout the day to prevent stomach compression on the heart.

Considering the potential vasovagal component in the syncope response, it is important to educate the patient on recognizing prodromal symptoms. The patient should be encouraged to lie down in a supine position to reduce the risk of traumatic falls and attenuate the syncopal episode. Other nonpharmacologic therapies include tilt-table training, which has been shown to significantly reduce recurrence, though compliance with the protocol is often low [16].

Pharmacological treatments are generally limited, but medications that may be considered include beta-blockers, disopyramide, scopolamine, theophylline, midodrine, and selective serotonin reuptake inhibitors [9].

## Conclusions

Although rare, syncope associated with hiatal hernia should be considered in patients with recurrent episodes, requiring a thorough evaluation with CT imaging and appropriate surgical or supportive management to prevent further complications. The purpose of this case discussion was to highlight that while syncope and hiatal hernias are both relatively common conditions in the general population, it is crucial to recognize the rare cause-and-effect relationship they may share. The limitations of this case report include the lack of a tilt-table test, which was not performed due to institutional constraints. This test could have helped pinpoint the exact etiology of the syncope, depending on the reproduction of symptoms. Another limitation was that the patient was lost to follow-up and did not retrieve her Holter monitor for evaluation.
